# Synthesis and photophysical studies of new fluorescent naphthalene chalcone

**DOI:** 10.1038/s41598-025-17566-1

**Published:** 2025-08-28

**Authors:** Raksha C. H., Yogeesh M., Rajeev K. Sinha, Nitinkumar S. Shetty

**Affiliations:** 1 Department of Chemistry, Manipal Institute of Technology, Manipal Academy of Higher Education, Manipal, 576104 Karnataka India; 2 Department of Physics, Birla Institute of Technology, Mesra, Ranchi, Jharkhand India

**Keywords:** Chalcone, Aldol condensation, Fluorescence, Photophysical studies, Chemistry, Materials science, Optics and photonics, Physics

## Abstract

This study reports the base-catalyzed Aldol condensation route for synthesizing a new naphthalene chalcone derivative (C1). The molecular structure of C1 was ascertained by FTIR, NMR (^1^H and ^13^C NMR) spectroscopy, and mass spectrometry. Optical properties were explored through UV-Visible absorption and photoluminescence spectroscopy. The UV-Vis spectrum indicated the presence of absorption bands from 300 to 350 nm due to π–π* transitions. In the solid state, C1 had a broad excitation that resulted in a solitary emission peak, whereas in solution, it emitted fluorescence at 495 nm. Density Functional Theory (DFT) calculations were carried out to investigate the electronic configuration and optical properties further. These findings suggest that the synthesized compound may possess favorable properties in optoelectronic and sensing applications.

## Introduction

**Fig. 1:**
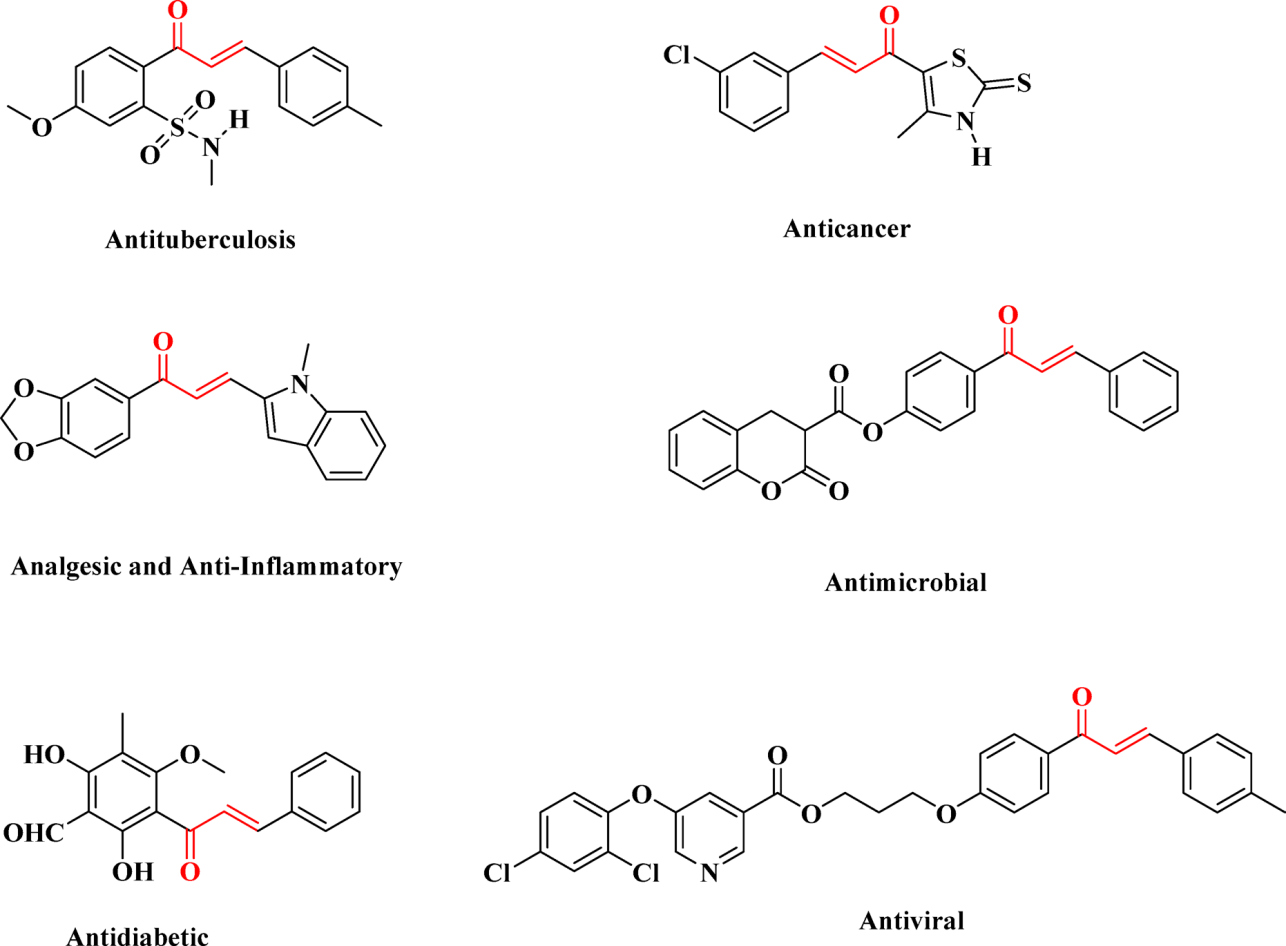
Structures of the representative chalcone derivatives exhibiting diverse biological activities.

Chalcones have gained significant attention in modern chemistry because of their broad spectrum of biological activities, structural versatility, and ease of chemical modification. A general chalcone structure consists of two aromatic rings linked by an α, β-unsaturated carbonyl bridge of three carbon atoms. A lot of studies have revealed that the incorporation of different heterocycles into the basic skeleton of chalcones drastically enhanced their pharmacological and material properties^1–5^. Chalcones are known to possess several pharmacological activities such as, antibacterial^6,7^, antifungal^6,8^, antimicrobial^8^, anti-inflammatory^8,9^, antimalarial^8–10^, anticancer^8–10^ and antioxidant^8,9,11^ Fig. 1. The position and type of substituents on the aromatic ring have an essential effect on the electron distribution and stability of the molecule. Electron donor or acceptor groups can significantly change chalcones’ reactivity and UV-Vis absorption properties^12,13^. The substituents also enhance the biological activity of chalcone compounds^14,15^. Chalcones are essential intermediates in the preparation of many biologically active heterocyclic compounds such as 1,4-diketones, benzothiazepines, flavones, and pyrazolines^8^.

Chalcone derivatives usually result from the interaction of aryl ketones with aromatic aldehydes in the presence of condensing agents. The aromatic rings in such compounds have a delocalized π-electron system^6^. Chalcones can be synthesized through various reactions, a few of which are Claisen-Schmidt Condensation^16,17^, Aldol Condensation^17,18^, Suzuki-Miyaura Coupling^19^, and Microwave-assisted synthesis^17^. Fluorescence chalcone derivatives have been widely applied in various fields due to their special photophysical characteristics, i.e., high fluorescence, tunable emission wavelengths, and remarkable absorption within the UV or visible region. This leads to valuable applications in the fields of bioimaging and diagnostic methods^20,21^, drug delivery^22^, detection of metal ions, anions, or small organic molecules via fluorescence quenching or enhancement^23^, fluorescent polymers^24^, optoelectronic devices^25^, DNA sensing^26^ and chromophoric probes^27,28^ etc. Several analytical methods are used to establish the’ structure, purity, and characterization of chalcones. UV spectroscopy verifies the 250–400 nm absorption range, corresponding to conjugated α, β-unsaturated systems. Infrared (IR) spectroscopy detects essential functional groups like carbonyl (C = O), alkene (C = C), and aromatic C–H bonds. Nuclear Magnetic Resonance (NMR) spectroscopy provides comprehensive structural information, with ^1^H NMR detecting signals of aromatic and α, β-unsaturated protons, whereas ^13^C NMR detects carbonyl and conjugated carbon atoms^29–32^. Mass spectrometry is predominantly used to confirm chalcone compounds’ molecular weight and study the fragmentation patterns. Thin-layer chromatography (TLC) techniques are commonly used to check reaction progress and contribute to the purification process. Combined analysis tools allow for better characterization, vital for different practical applications. It is essential to develop a thorough comprehension of photophysical properties to understand molecular interactions with light, which is central to the progression of technologies in energy, healthcare, and materials science. UV-Visible and fluorescence spectroscopy are two of the primary methods used to measure light absorption and emission, providing information regarding the optical activity of chalcones. Besides, research involving Density Functional Theory (DFT) and photoluminescence gives more insight into molecular structure-related optical performance. Based on these unique characteristics, chalcones have emerged as immensely useful in light-harvesting devices, photonic materials, fluorescent sensors^33^, and bioimaging applications.

## Experimental

### Materials and methods

All chemicals utilised for synthesis and characterization were of analytical grade, purchased from commercial suppliers, and used without additional purification.

#### Analytical and spectroscopic characterization

Thin-layer chromatography (TLC) was used to evaluate the purity of the compound, which was further confirmed by measuring its melting point using an open capillary technique with Thiele’s tube apparatus. Attenuated Total Reflectance Fourier Transform Infrared (ATR-FTIR) spectroscopy was employed for solid-state characterisation, utilising a Shimadzu spectrometer with spectra recorded at room temperature in the range 400–4000 cm^−1^ using 40 scans at 4 cm^−1^ resolution. A Bruker instrument was used to record NMR spectra at 400 MHz for ^1^H and 100 MHz for ^13^C nuclei with deuterated dimethyl sulfoxide (DMSO-d_6_) as solvent and tetramethylsilane (TMS) serving as internal standard. The Shimadzu UV-3600 spectrophotometer was used to record the Ultraviolet-Visible (UV-Vis) absorption spectra of 0.01 mM solutions in dimethylformamide (DMF), using quartz cuvettes with a 1 cm pathlength, using DMF as reference solvent. Fluorescence measurements were performed at room temperature with a Jasco FP-8300 spectrofluorometer with a 450 W xenon lamp for solid and solution states. The solution samples were prepared at a concentration of 0.1 mM.

#### Density functional theory (DFT) calculations

Density functional theory (DFT) was used to understand the structure and spectroscopic characteristics of the molecule. Structure of the molecule was optimized in the ground (S_0_) and first electronic excited state (S_1_). For the ground state optimization, the Becke-3- Lee-Yang-Parr (B3LYP) functional with TZVP basis set was utilized. The B3LYP functional was successful in the interpretation of chalcones used in our earlier investigations^2,4^. The time-dependent B3LYP (TD-B3LYP) method was used for the calculations in the first excited state. Harmonic frequency calculation was also performed in the ground state to ensure the optimized structure was at the global minimum for the potential energy surface. Vertical excitation energies from the optimized ground state structure to various excited states were calculated to understand the molecule’s absorption spectrum (UV-Vis). The TD-B3LYP functional was used for the absorption spectrum calculation. Both geometry optimization and absorption spectrum calculation were performed in vacuum and dimethylformamide (DMF) solvent environments. All the DFT calculations were accomplished using the Gaussian09 programme suite^34^.

### Synthesis of (E)−3-(1-methoxynaphthalen-2-yl)−1-(6-methoxynaphthalen-2-yl) prop-2-en-1-one (C1)

An equimolar composition (0.1 g each) of 6-methoxy-2-acetonaphthanone and 1-methoxy-2-naphthaldehyde was dissolved in ethanol. A 40% potassium hydroxide (KOH) solution was added dropwise while stirring at room temperature. The reaction mixture was stirred at room temperature for 10–12 h, and its progress was traced with the help of thin-layer chromatography (TLC). Crushed ice was then added to the mixture, and the solid obtained was neutralized and filtered. Recrystallization was carried out using hot ethanol.

### Analytical and spectral data

Pale orange crystalline solid; yield: 90%; mp: 163–166 °C; FT-IR (ν max, cm^−1^): 2961 (C-H stretch), 1651 (C = O stretch), 1585 (aromatic C = C stretch), 1061 (C-O stretch); ^1^H NMR (400 MHz, DMSO-d_6_): δ 8.73 (s, 1H, HC = CH), 8.41–8.37 (d, 1H, Ar-H), 8.26–8.24 (d, 1H, Ar-H), 8.13–8.07 (m, 4 H, Ar-H), 7.98–7.95 (d, 2 H, Ar-H), 7.64–7.57 (m, 2 H, Ar-H), 7.47–7.44 (d, 2 H, Ar-H), 7.29–7.26 (d, 1H, Ar-H), 4.09 (s, 3 H, OCH_3_), 3.93 (s, 3 H, OCH_3_); ^13^C NMR (100 MHz, DMSO-d_6_): δ 189.56, 159.92, 157.51, 137.36, 136.87, 133.52, 132.70, 132.60, 131.89, 130.52, 129.28, 129.09, 128.33, 128.05, 127.83, 127.26, 125.28, 124.37, 123.43, 119.96, 116.73, 113.90, 106.55, 56.97, 55.91; Molecular formula: [C_25_H_20_O_3_]. HRMS mass (m/z): 369.15[M + H] ^+^.

**Fig. 2:**
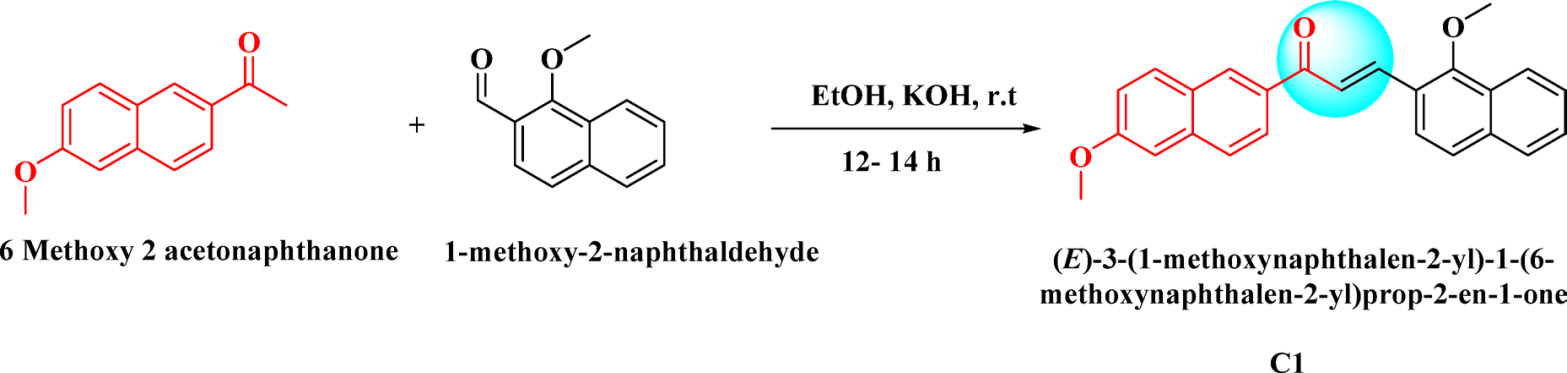
Synthetic scheme for (E)−3-(1-methoxynaphthalen-2-yl)−1-(6-methoxynaphthalen-2-yl) prop-2-en-1-one (C1), synthesized via a base-catalysed Claisen–Schmidt condensation of 1-methoxy-2-acetylnaphthalene and 6-methoxy-2-naphthaldehyde.

## Results and discussion

### Chemistry

**Fig. 3:**
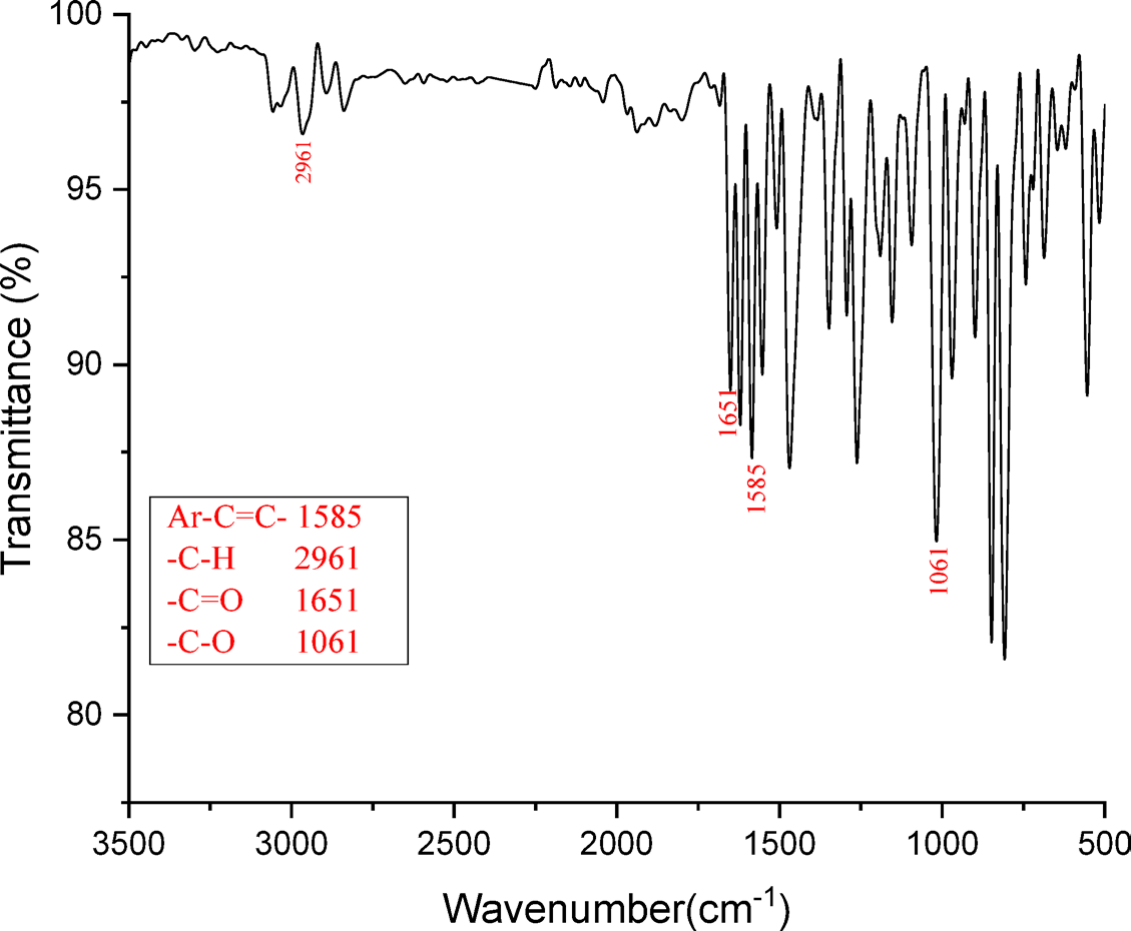
FTIR spectrum of (E)−3-(1-methoxynaphthalen-2-yl)−1-(6-methoxynaphthalen-2-yl)prop-2-en-1-one (C1).

**Fig. 4:**
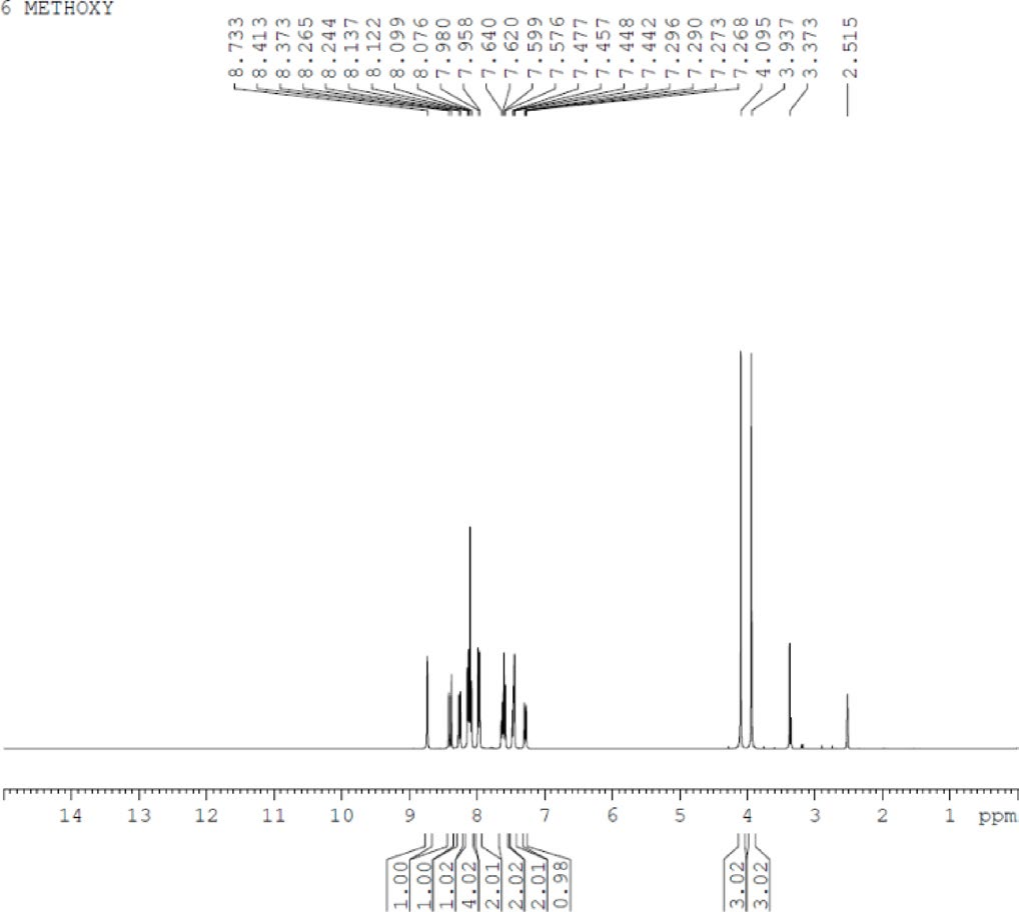
¹H NMR spectrum of (E)−3-(1-methoxynaphthalen-2-yl)−1-(6-methoxynaphthalen-2-yl)prop-2-en-1-one (C1) recorded in DMSO-d₆.

**Fig. 5:**
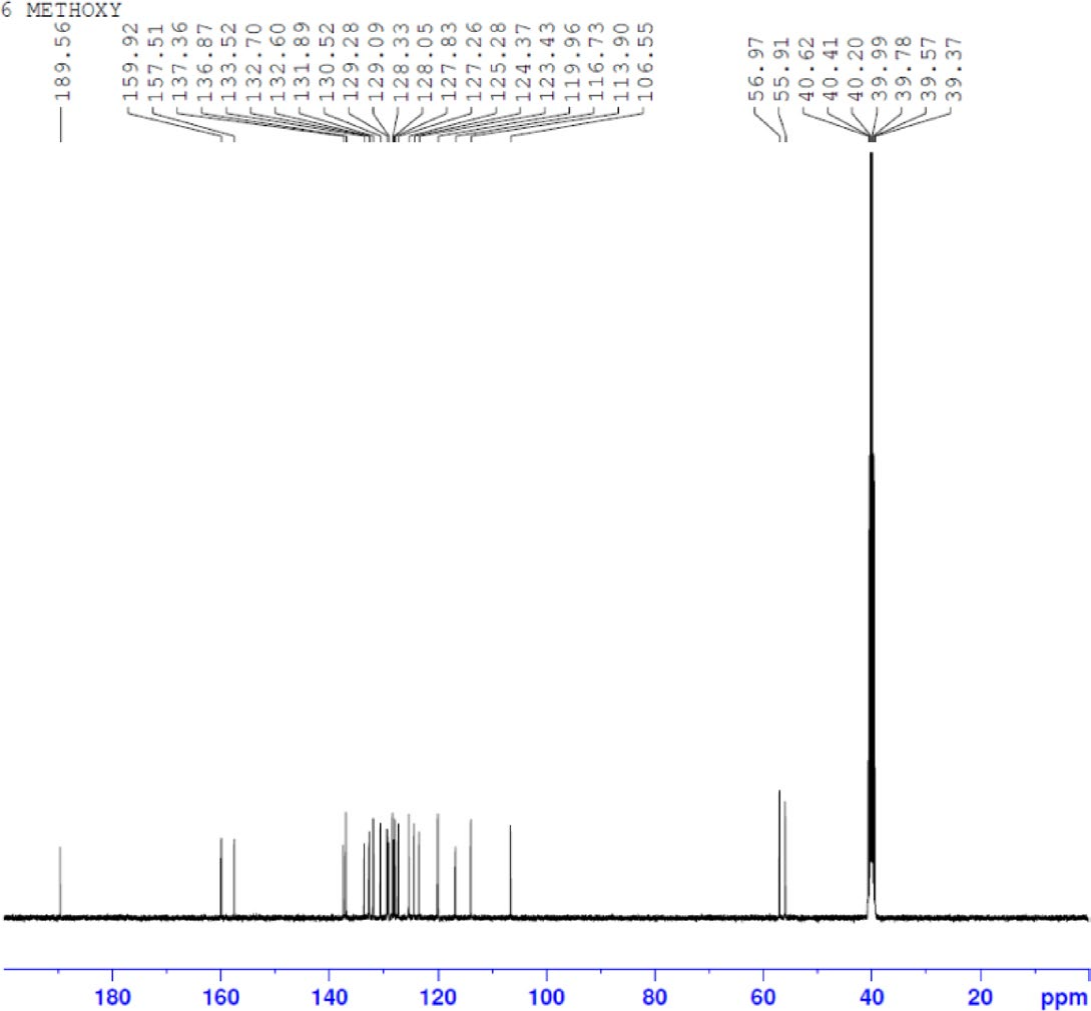
¹³C NMR spectrum of (E)−3-(1-methoxynaphthalen-2-yl)−1-(6-methoxynaphthalen-2-yl)prop-2-en-1-one (C1) in DMSO-d₆.

**Fig. 6:**
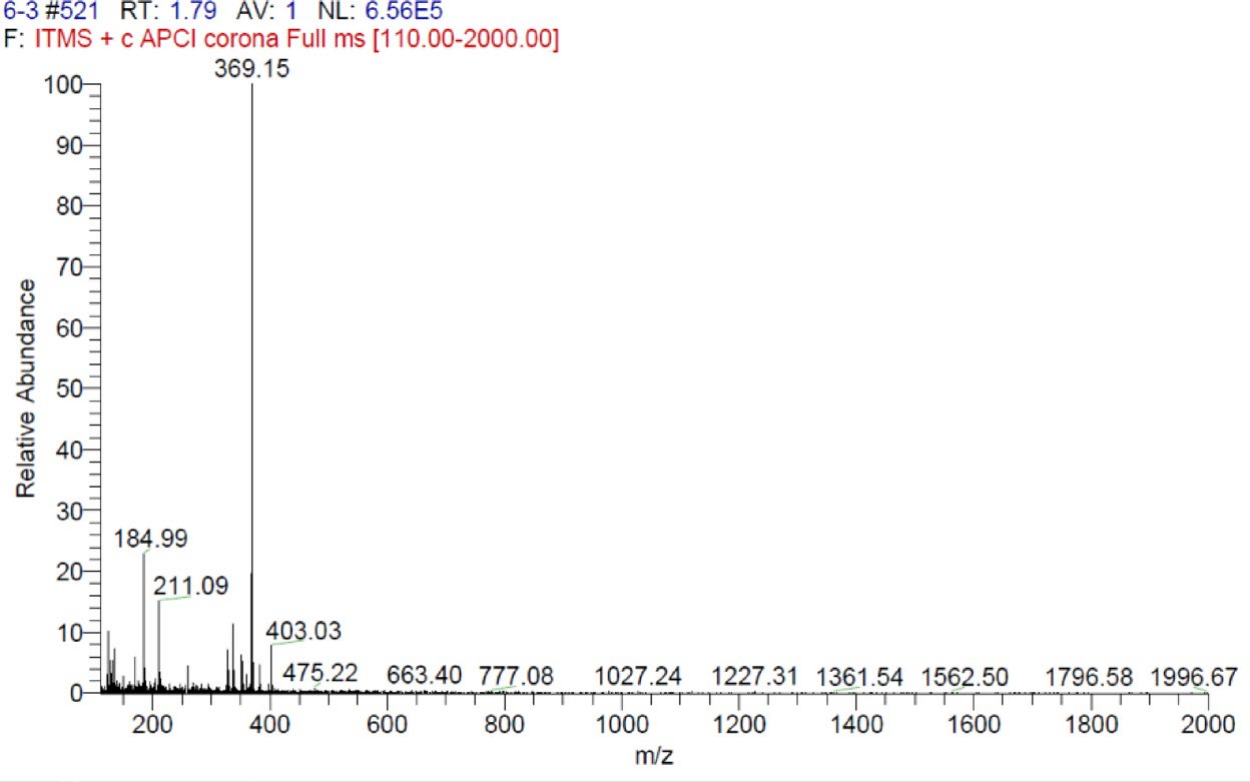
Mass spectrum of (E)−3-(1-methoxynaphthalen-2-yl)−1-(6-methoxynaphthalen-2-yl)prop-2-en-1-one (C1) which revealed a molecular ion peak at m/z 369.15, corresponding to the protonated molecular ion [M + H]⁺ of C1.

A naphthalene-based chalcone derivative was successfully synthesized via a conventional base-catalyzed Aldol condensation reaction, employing equimolar quantities of an aromatic aldehyde and ketone in ethanol as the solvent, as depicted in Fig. 2^4^. This method facilitated the formation of the α, β-unsaturated carbonyl system, which serves as a key structural motif in chalcones. To confirm the structure of the synthesized compound, a comprehensive spectral characterization was conducted using FTIR, ^1^H NMR, ^13^C NMR, and mass spectrometry techniques. The FTIR spectrum exhibited several diagnostic absorption bands that confirmed the formation of the chalcone framework. A strong band at 1651 cm^−^¹ was attributed to the stretching vibration of the conjugated carbonyl group (C = O), indicative of the enone system. An aromatic C = C stretching band at 1585 cm^−^¹ and a C–O stretching band at 1061 cm^−^¹ further validated the α, β-unsaturated carbonyl moiety (Fig. 3). ^1^H NMR spectroscopy provided further structural insight. The spectrum revealed a singlet at δ 8.73 ppm corresponding to the vinylic proton adjacent to the carbonyl group, confirming the formation of the conjugated double bond. Multiplets in the aromatic region confirmed the presence of the aromatic protons. Notably, singlets observed at δ 4.09 and δ 3.93 ppm were assigned to methoxy (-OCH₃) substituents attached to the aromatic rings (Fig. 4). The ^13^C NMR spectrum supported these findings by displaying a prominent downfield signal at δ 189.56 ppm, characteristic of a carbonyl carbon in a conjugated system. Additional signals in the spectrum corresponded to the various aromatic carbons, and two distinct signals at δ 55.95 and δ 56.97 ppm confirmed the presence of two methoxy-substituted carbon atoms, further supporting the substitution pattern on the aromatic system (Fig. 5). Mass spectrometric analysis revealed a molecular ion peak at m/z 369.15, corresponding to the compound’s protonated molecular ion [M + H]⁺. This value is in excellent agreement with the calculated molecular weight for the molecular formula C₂₅H₂₀O₃, further confirming the target chalcone derivatives successful synthesis and structural integrity (Fig. 6).

### Density functional theory (DFT) calculations

**Fig. 7:**
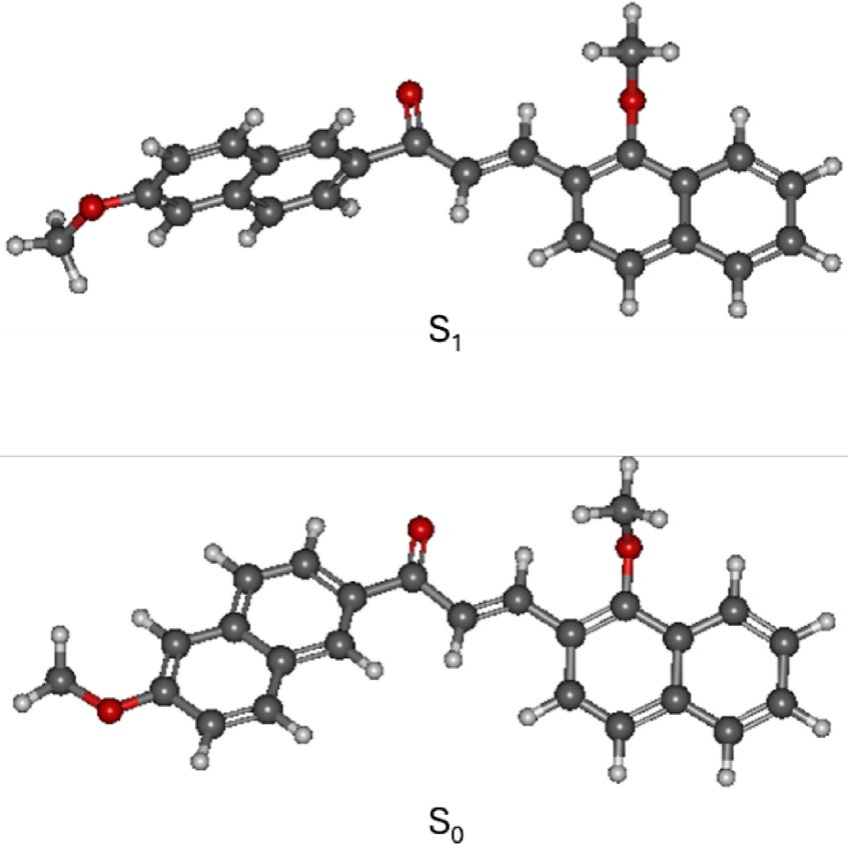
Optimized structures of C1 in ground (S_0_) and first excited (S_1_) states. Optimizations in S_0_ and S_1_ were performed using B3LYP/TZVP and TD-B3LYP/TZVP levels, respectively.

DFT calculations were also performed to corroborate the FTIR, NMR, and mass spectrometry results on the molecule’s structure. The structure of the molecule in the ground and excited states was optimized, followed by frequency calculation and frontier molecular orbital analyses. Optimized molecular geometries of the compound in its ground (S_0_) and first excited (S_1_) electronic states are presented in Fig. 7. It is found that in the ground state, the structure is nearly planar with the napthalene ring adjacent to the keto group out of plane by 13 degrees relative to the another napthalene ring. The electronic excitation to the S_1_ state causes strong conformational changes with a large torsional twist between the middle aromatic moiety and the outer aryl rings. As a result, the napthalene ring adjacent to the keto group goes out-of-plane by 96 degrees. Optimization in solvent DMF does not generate appreciable changes. The relative tilt of the napthalene ring in the ground and excited states was 15 and 101 degrees, respectively. The near-planar structure in the ground state evidences an extended π-conjugated system, which decreases upon electronic excitation, presumably caused by intramolecular charge transfer (ICT). Also, subtle alterations in the positions of the methoxy and methyl substituents are noted, which can slightly influence the electron distribution in the π-framework.

**Fig. 8:**
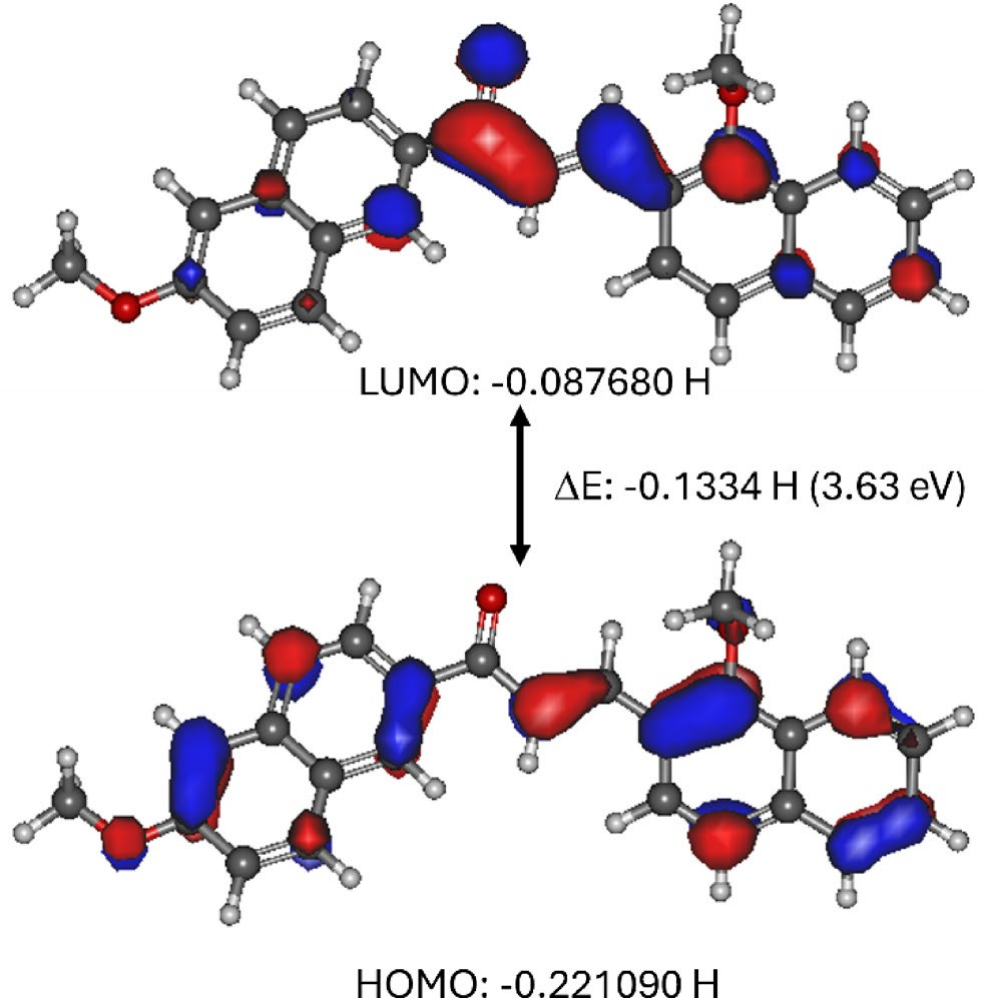
HOMO and LUMO plots with energy gap for the optimized structure of compound C1. The HOMO, with π-symmetry, shows electron density primarily on the dimethoxyphenyl ring and the ketoethylenic bridge, indicating electron-donating regions.

The frontier molecular orbitals, namely the HOMO and LUMO, were analyzed to understand the observed structural changes in the molecule. The HOMO and LUMO analysis is also essential as it yields valuable information regarding the electronic distribution, optical properties, and general stability of organic molecules, especially those that will be used for optoelectronic devices. Figure 8 shows the HOMO and LUMO plots with the energy difference between them. As it is evident from the figure, the HOMO has π-symmetry with the electron density restricted mainly to the dimethoxyphenyl ring and ketoethylenic segment, signifying their role as electron-donating sites. Conversely, the LUMO (π*-symmetry) is predominantly localized on the carbonyl and naphthyl moieties, which are electron acceptors, defining a preferred path for electron transfer upon excitation and facilitating an effective intramolecular charge transfer process. The observed electron densities in HOMO and LUMO explain the observed reorganization in the molecular structure upon electronic excitation. The calculated energy gap between the HOMO and LUMO is found to be 0.1334 H (3.63 eV; ~341 nm), leading to the possibility of absorption close to the UV region (discussed in the next section). In the DMF, the calculated HOMO to LUMO energy gap is 0.12776 H (3.476 eV; ~357 nm). The efficient charge-transfer ability and high molecular polarizability make the compound a good candidate for applications such as organic solar cells, OLEDs, and fluorescence sensors.

### Photophysical properties

#### UV-Vis spectral analysis

**Fig. 9:**
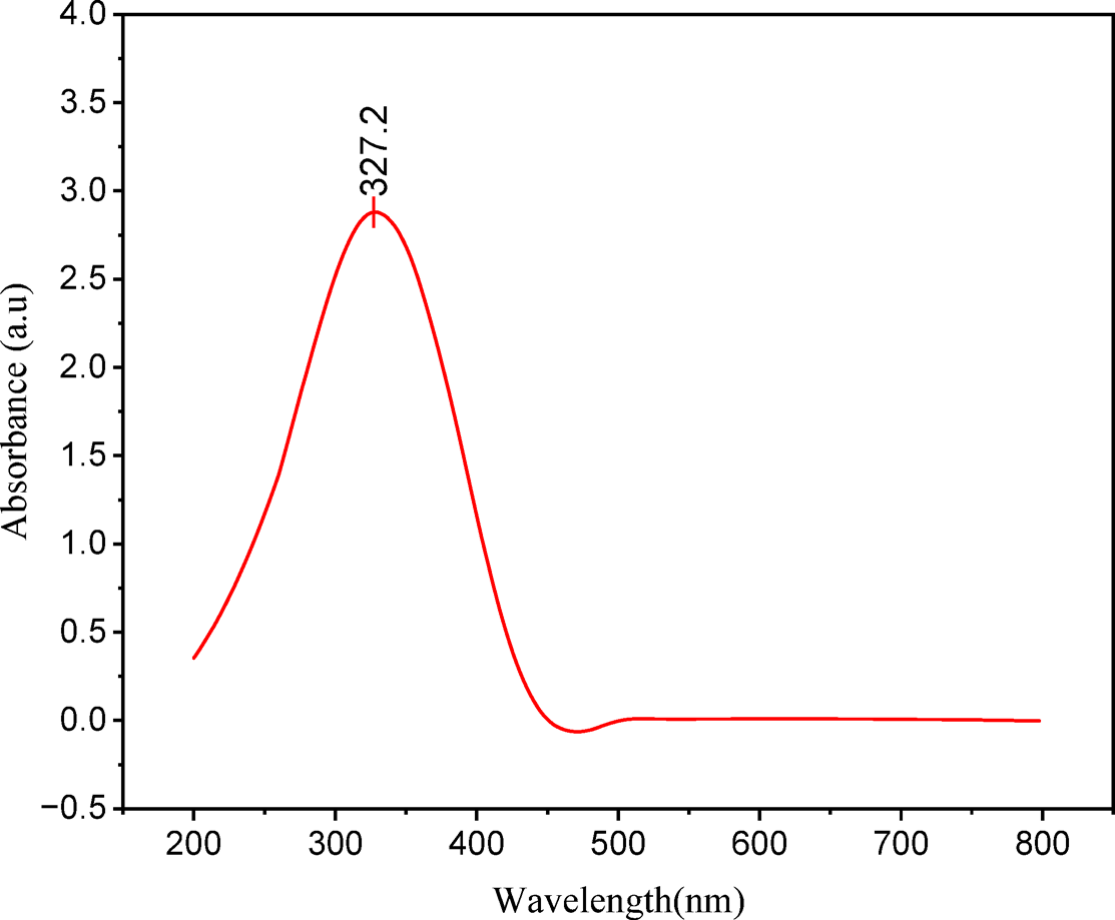
Experimental UV–Vis absorption spectrum of compound C1 recorded in DMF. The spectrum shows a strong absorption band with a maximum at 327.2 nm, attributed to a π–π* transition associated with the conjugated system of the molecule, indicating extended electron delocalization.

Figure 9 shows the UV-Vis spectrum, where an intense absorption band in the ultraviolet region was observed. The absorption maximum is observed at 327.2 nm. The observed band maximum position is in excellent agreement with the HOMO to LUMO energy gap (341 nm for the molecule without solvent and 356 nm for the molecule in DMF environment) discussed above. The observed absorption is typical of a π→π* transition, which is typical in conjugated system compounds. The occurrence of this peak confirms the presence of a conjugated structure in C1, possibly with delocalized π-electrons.

**Fig. 10:**
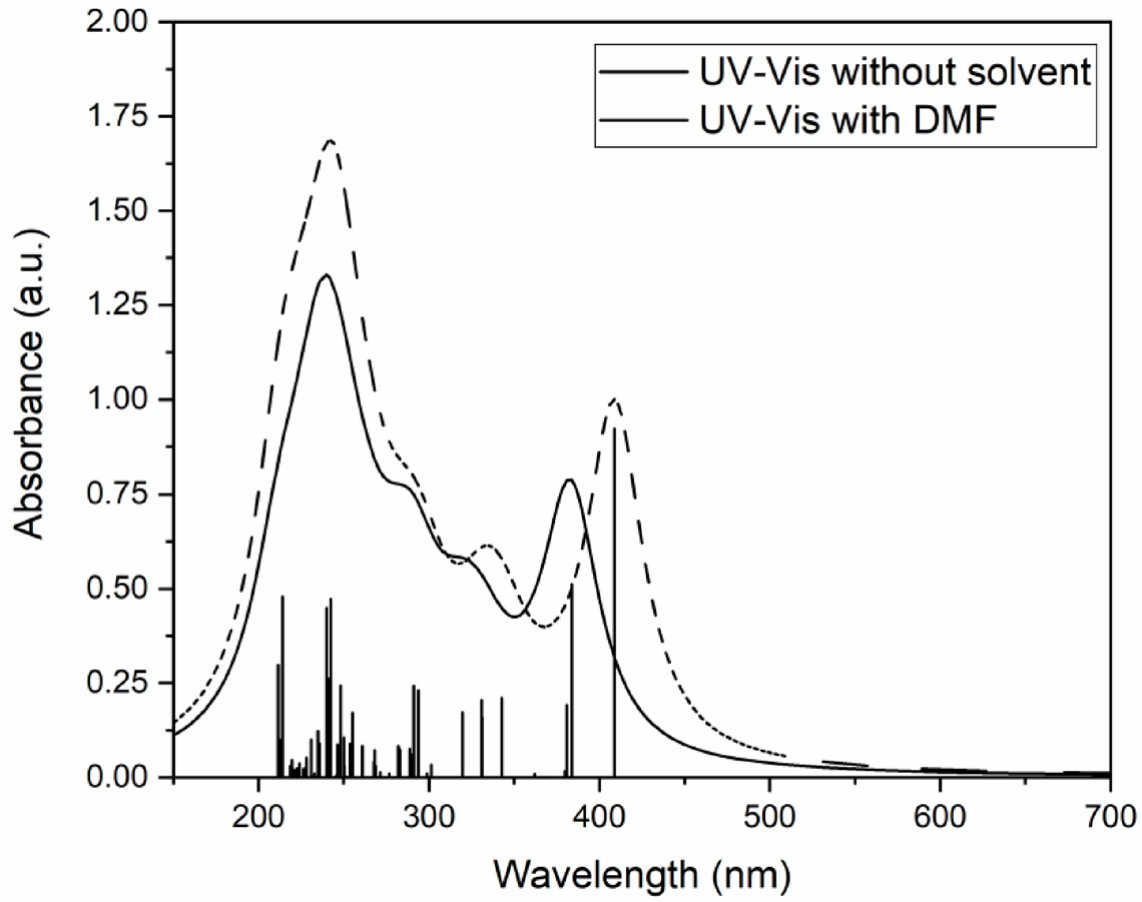
Calculated absorption spectra of compound C1 using TD-B3LYP/TZVP level of theory, with and without DMF solvent.

To understand the observed UV-Vis spectrum, the UV-Vis absorption spectrum of the molecule with and without DMF solvent was calculated using TD-B3LYP theory and TZVP basis set. In the calculation, vertical excitation energies of thirty excited states were calculated. Figure 10 shows the calculated absorption spectra with and without DMF. In the spectrum without DMF, the first excited state is observed at 384 nm with maximum oscillator strength (0.5117). The calculated band maximum is on the red side compared to the HOMO-LUMO energy gap. It is found that though the major contribution to the excitation to the S1 state is from the HOMO-LUMO transition, some contribution is also from the HOMO-2→LUMO and HOMO-4→LUMO, which may have caused the calculated red shift. The basis set (TZVP) used in the calculation may also be responsible for the observed red shift, which might have underestimated the calculated energy gap. A similar situation was found for the molecule under the DMF environment, where the HOMO-LUMO energy gap is 357 nm and the absorption spectrum maximum is 409 nm. Compared to the experimentally observed absorption spectrum, the band maximum is red shifted by ~ 82 nm. Such an appreciable red shift could be due to the used basis set and the solvent. The used solvation method (SCRF) uses the solvent as dielectric continuum around the solute molecule and therefore it may not consider the specific molecular interaction between solute and solvent molecules, which may be responsible for the observed red shift.

#### Fluorescence spectral analysis

##### Solid-state fluorescence

**Fig. 11:**
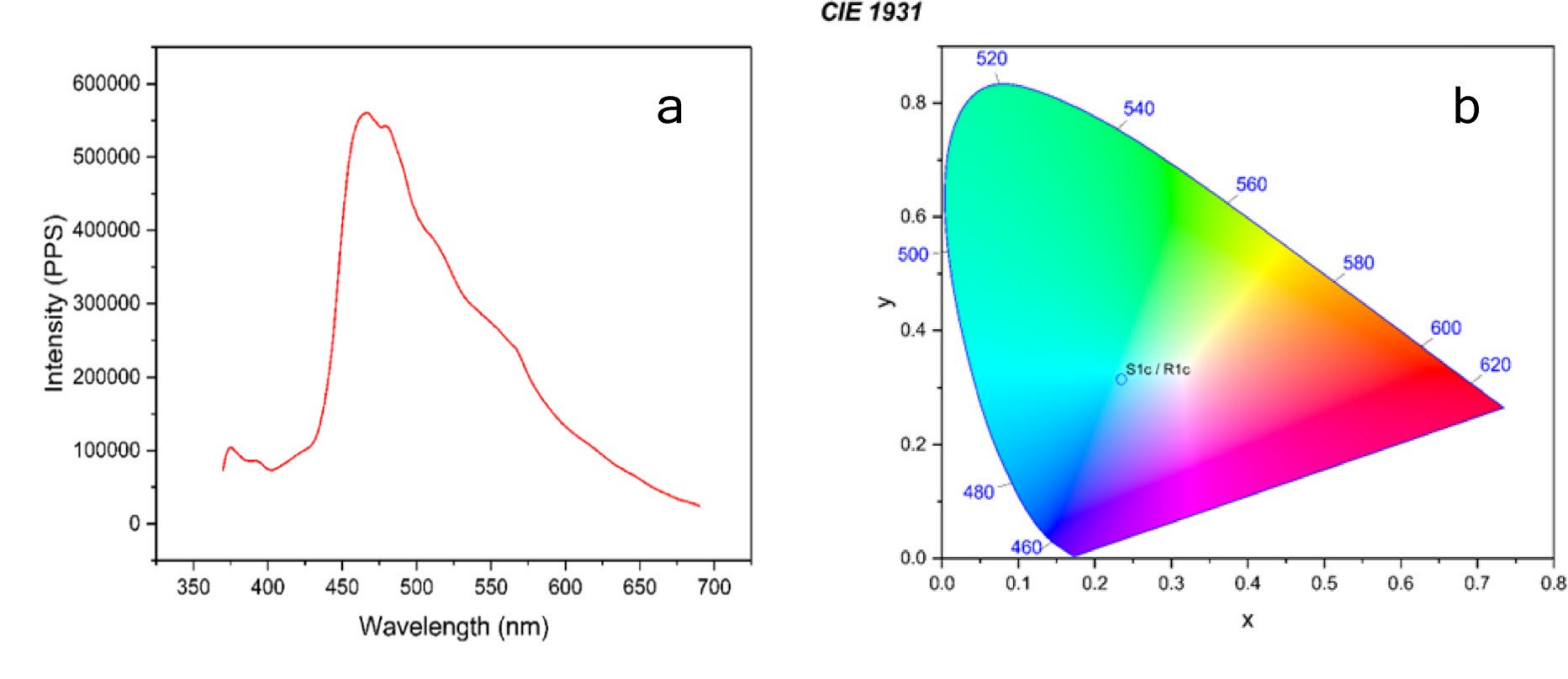
(**a**) Two-dimensional fluorescence spectrum of C1 at λex = 467 nm. (**b**) CIE coordinates of solid-state emission.

Compound C1 shows strong photoluminescence in the solid state with a prominent peak at 467 nm, corresponding to the blue region of the visible spectrum Fig. 11 (a & b). This fluorescence comes mainly from an intramolecular charge transfer (ICT) process. A weak tail toward 700 nm reflects the occurrence of lower-energy transitions, possibly due to molecular packing or structural defects in the solid. The CIE 1931 colorimetric chart shows the emitted light in the greenish-cyan region. Regardless of the usual quenching effects resulting from molecular aggregation, the vivid solid-state emission indicates either decreased aggregation-caused quenching (ACQ) or the existence of aggregation-induced emission (AIE) behavior. These characteristics suggest the compound’s suitability for application in solid-state optoelectronic devices.

##### Solution-state fluorescence

**Fig. 12:**
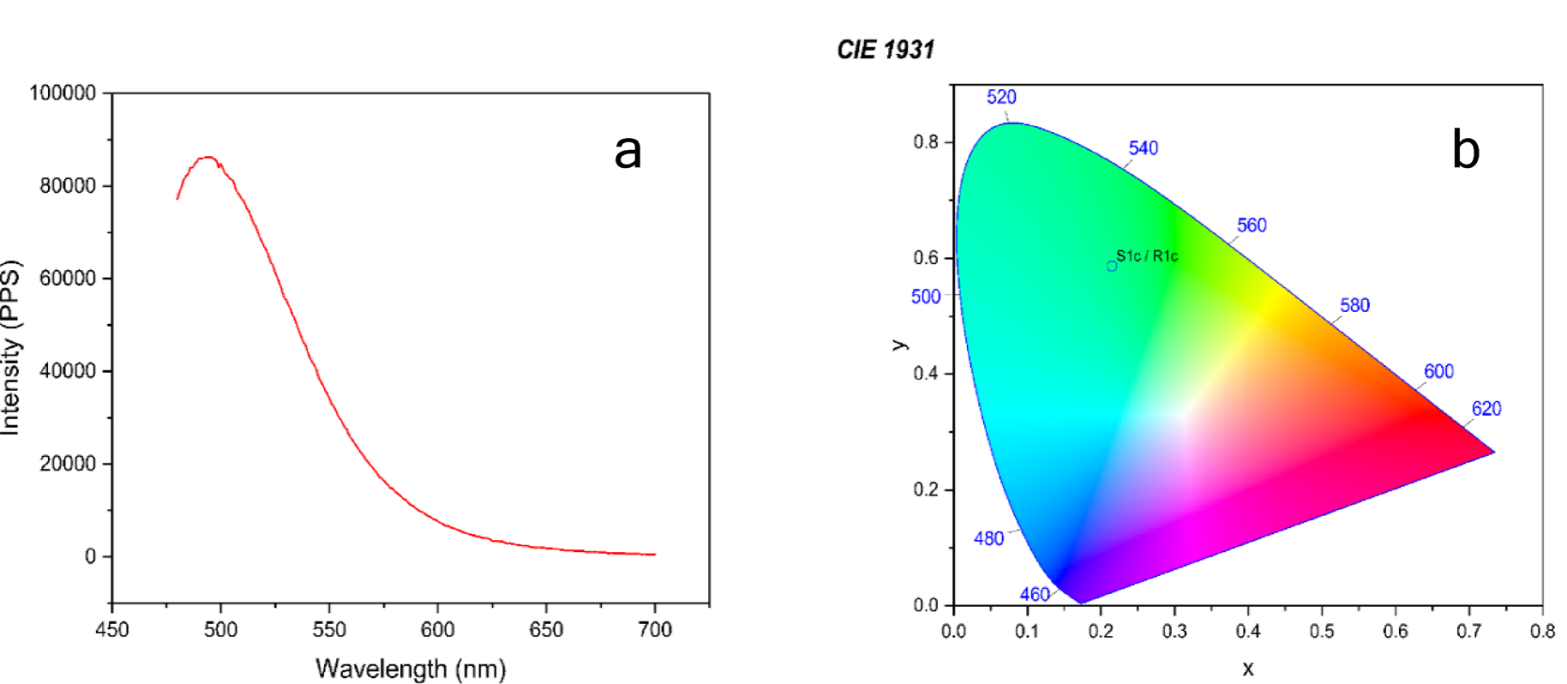
(**a**) Two-dimensional fluorescence emission spectrum of compound C1 in DMSO at λex = 495 nm. (**b**) CIE 1931 chromaticity diagram showing solution-state emission.

Photoluminescence (PL) spectroscopy, a non-invasive analytical technique, was employed to explore the luminescent behavior of the material. The emission characteristics of the synthesized chalcone in solution were examined using DMSO. Solvated in solution, the compound fluoresces at 495 nm, which is in the cyan-blue region of the spectrum Fig. 12 (a & b). This fluorescence, as in the case of intramolecular charge transfer (ICT) transitions, seems to be enhanced by the polarity of the solvent, possibly through stabilization of the excited state. The weak emission tail that extends from 700 nm suggests the presence of other low-energy emissive states. The chromaticity coordinates position the emission in the region of cyan on the CIE diagram. Such behavior indicates successful interaction with the solvent as well as with the molecule, also structural mobility in solution attributes that recommend its use in solution-processable optical devices. The material is highly photoluminescent and responsive to its surroundings, both in the solid and the solution state. A red shift is seen, progressing from blue emission (~ 467 nm) in solid to cyan-blue (~ 495 nm) in solution, which mirrors ICT-enabled fluorescence and solvent interactions. Due to the significant emission in the solid state and increased fluorescence in solution, the synthesized compound may be a optimistic candidate for application in optical devices like OLEDs, sensors, and flexible light-emitting devices. The compound (*E*)−3-(1-methoxynaphthalen-2-yl)−1-(6-methoxynaphthalen-2-yl)prop-2-en-1-one (C1) had intense emission at 0.1 mM concentration with a significant quantum efficiency of 22.62%.

## Conclusion

In conclusion, we effectively synthesized a new fluorescent naphthalene chalcone molecule (C1) via base-catalyzed Aldol condensation. We confirmed the structures of the synthesized compounds through FTIR, ¹H NMR, ¹³C NMR, and mass analyses. The DFT studies revealed notable conformational changes and intramolecular charge transfer upon excitation, supported by HOMO–LUMO analysis. The near-planar geometry in the ground state and the twisted structure in the excited state indicate a loss of π-conjugation due to electronic excitation. The electron density distribution suggests efficient donor–acceptor interaction within the molecule. The photochemical behavior of C1 was investigated and has shown interesting results. The chalcone C1 showed emission at 495 nm and exhibited solid-state emission in the greenish-cyan region. These findings may underscore the compound’s suitability for optoelectronic applications such as OLEDs, organic solar cells, and fluorescence-based sensors. In alignment with SDG 7 (Affordable and Clean Energy), the development of photoluminescent material contributes to advancing low-energy-consumption technologies in display technologies and sensing, supporting a more sustainable energy future.

## Data Availability

Data is provided within the manuscript.
